# Long‐Lifespan Polyorganosiloxane‐Based Silicone Evaporator for High‐Efficiency Seawater Desalination

**DOI:** 10.1002/advs.78278

**Published:** 2026-10-14

**Authors:** Jiaxian Zheng, Liqiang Zhang, Haoqi Ni, Shuozhen Chen, Xiangfeng Lin, Liwei Wang, Zhanhui Yuan

**Affiliations:** ^1^ College of Materials Engineering Fujian Agriculture and Forestry University Fuzhou People's Republic of China; ^2^ College of Materials and Chemical Engineering Minjiang University Fuzhou Fujian China

**Keywords:** desalination, evaporation, materials science, solar desalination

## Abstract

Conventional evaporators suffer from poor mechanical strength, salt crystallization, and degradation in sea water. We design a white polyorganosiloxane‐based solar evaporator (PDMSE) that exploits siliconeʼs unique attributes. These include chemical inertness, anti‑corrosion, high flexibility, and chemical resistance. Such characteristics ensure structural integrity and anti‑fouling performance under harsh brine conditions. The white surface combined with a reflector enables efficient light scattering and heat localization. Reflector‐equipped PDMSE achieves evaporation rates of 4.344 kg m^−2^ h^−1^ at 1 sun and 8.087 kg m^−2^ h^−1^ at 3 suns in 3.5 wt.% NaCl. It shows no salt accumulation over 8 h operation and maintains stable cycling for 53 days. Moreover, PDMSE withstands seawater immersion for 197 days without degradation. The evaporator also purifies sea water to World Health Organization drinking standards. This work provides a durable and scalable silicone‑based strategy for high‑performance desalination.

## Graphical abstract

Conventional evaporators suffer from salt crystallization and degradation. We design a white polyorganosiloxane evaporator with a reflector, achieving 4.344 kg m^−2^ h^−1^ at 1 sun in 3.5 wt.% NaCl. It shows over 53‑day cycling stability and 197‑day seawater resistance. The produced water meets WHO standards, providing a durable desalination strategy.

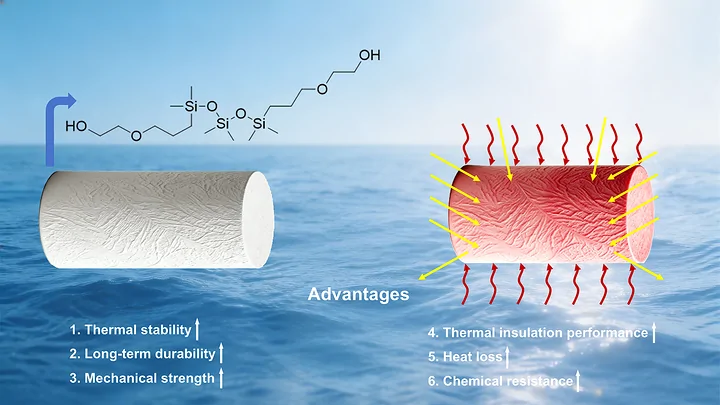

## 1 Introduction

Oceans cover 70% of Earth, offering abundant seawater for desalination [1]. Yet traditional methods like reverse osmosis and multi‐stage flash distillation are limited by high energy use, cost, and pollution [2]. Solar‐driven interfacial evaporation solves this by using photothermal materials to localize heat at the water‐air interface, enabling low‐energy, safe phase‐change evaporation [3, 4]. A typical evaporator has a photothermal layer, water transport layer, and vapor channel, with performance tied to conversion efficiency and lifespan [5, 6]. However, it is still difficult to design the stable evaporator that can exhibit long lifespan on the complex marine environment [7, 8].

The evaporator surface needs to maintain a sufficiently high temperature to drive water evaporation [9]. Black materials such as carbon black and graphite absorb sunlight and heat up quickly [10]. In actual seawater conditions, heat is lost to the surrounding air. Seawater convection also carries away thermal energy [11]. These effects reduce the evaporation rate. White photothermal materials can reflect sunlight. Therefore, they can enhance local photothermal conversion through internal scattering. This extends the optical path and improves solar energy utilization. An ideal evaporator material should resist radiation and degradation. It should also maintain structural integrity under wave impact. These properties affect the service life and long‑term stability of the evaporator. Biomass materials like wood and seaweed are prone to decay. They also have poor mechanical strength. They are not suitable for long‑term use in marine environments [12, 13, 14, 15, 16]. Designing a material with both stability and durability remains a challenge.

Polyorganosiloxane, with a backbone of siloxane bonds, is renowned for its high elasticity, low modulus, excellent flexibility, weather resistance, high thermal insulation effect, chemical stability, and biocompatibility [17]. Importantly, it inherently resists salt‑fog corrosion and microbial degradation, offering a long operational lifespan. These properties make silicone exceptionally suitable for the complex ocean environment, where it can tolerate severe wave action and mechanical stress. Moreover, silicone's low surface energy prevents salt adhesion, its tunable porosity enables efficient water transport and thermal insulation, and its high tear resistance ensures durability against repeated wave impacts. Unlike biomass materials, silicone is UV‐resistant and ozone‐resistant, maintaining stable performance under prolonged sunlight. It can be easily processed via molding or 3D printing for scalable, low‐cost production. Collectively, these features demonstrate silicone's universal applicability as a robust, versatile platform for solar‐driven seawater evaporators in ocean [18].

Despite these promising attributes, the long‑term service durability of the evaporator matrix itself, as opposed to that of the photothermal coating or the water‑transport layer, has received comparatively little attention in the existing literature. Most reported solar evaporators prioritize short‐term evaporation rate enhancement, while systematic investigations into the structural and chemical stability of the evaporator body under prolonged seawater exposure, cyclic thermal stress, and salt‐fog corrosion remain scarce [19]. For instance, biomass‐derived substrates such as wood and seaweed are prone to biological decay and mechanical degradation in marine environments, whereas certain hydrogel‐based evaporators suffer from swelling‐induced structural collapse or performance decay over extended operation [20]. Such limitations highlight the gap between laboratory‐scale demonstration and practical long‐term deployment in marine environments. This work addresses this gap by exploiting the intrinsic chemical inertness, corrosion resistance, and UV stability of the polyorganosiloxane network, targeting the evaporator matrix itself as the component for long‐term durability optimization, rather than focusing solely on photothermal conversion efficiency or salt‐rejection capability.

In this study, we designed a white polyorganosiloxane solar interfacial evaporator (PDMSE). The strong Si‐O backbone endows the material with exceptional thermal stability, chemical resistance, and long‐term durability in high‐salinity brines (Figure 1a). Its low thermal conductivity suppresses heat loss to bulk water, while its high hydrophobicity. and flexibility allow the construction of a self‐supporting three‐dimensional porous skeleton. As shown in Figure 1b, the 3D columnar structure not only enables light absorption by the material itself but also induces strong light scattering. With an external reflector, secondary optical utilization is achieved, and the corresponding heat flux (Figure 1c,d) reveals a concentrated photothermal effect on the sidewalls, creating a uniform 3D thermal field without localized cooling. Figure 1e illustrates the internal and surface water transport network, where rapid capillary pumping and convective pore flow ensure water transport, salt rejection, and stable operation in brine. The PDMSE delivered an evaporation rate of 3.285 kg m^−2^ h^−1^ with a solar‐vapor conversion efficiency of 90% in 3.5 wt.% NaCl solution, operated steadily for over 53 days, and remained intact in seawater for more than 197 days. It also efficiently purified organic dye wastewater, yielding freshwater that meets World Health Organization (WHO) drinking standards. This work offers a new strategy for scalable and distributed freshwater production with ultrahigh salt tolerance and high performance.

**FIGURE 1:**
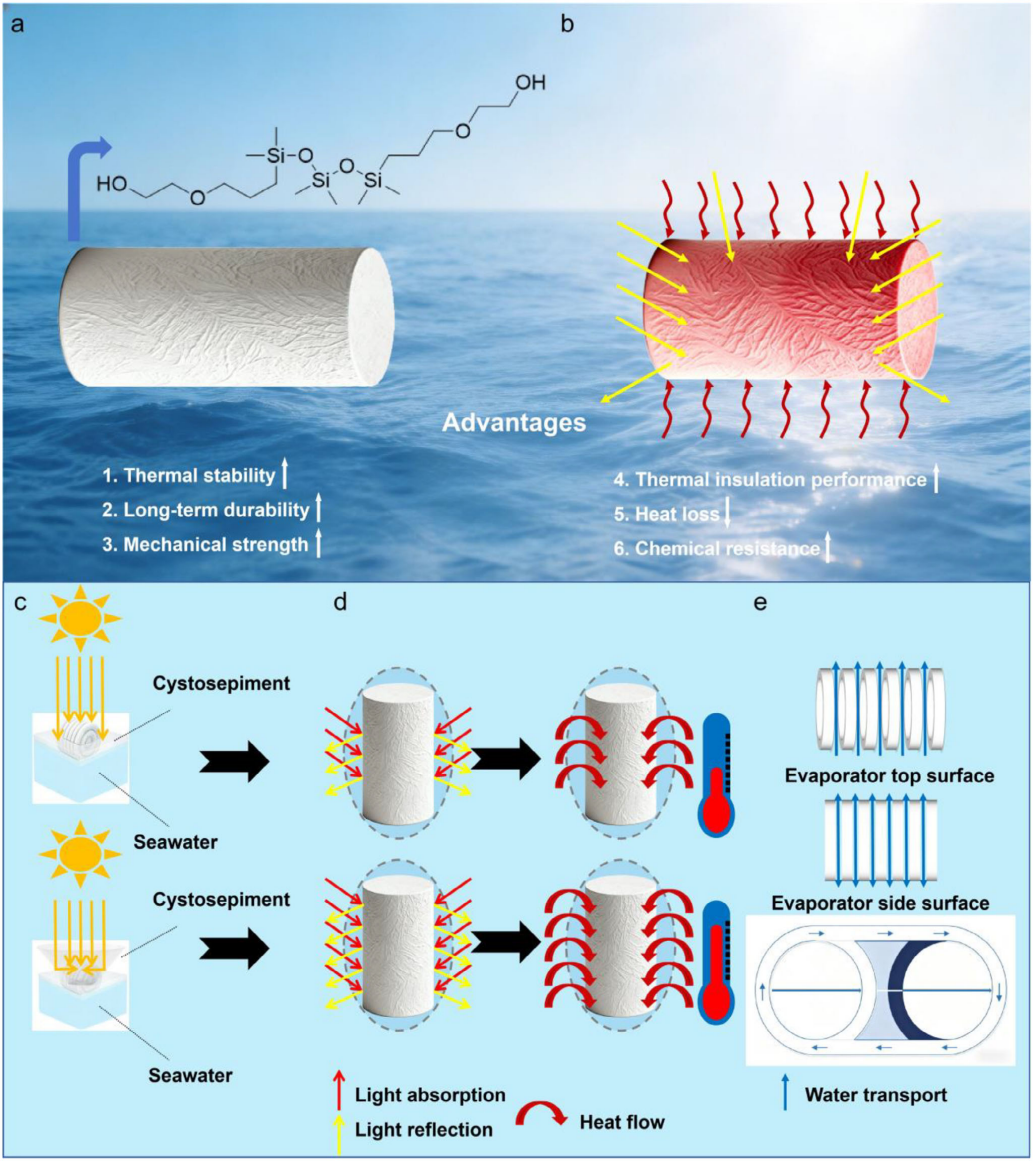
(a) Microscale hierarchical structure of the polyorganosiloxane matrix. (b) Light scattering and absorption in the 3D columnar geometry. (c) Overall evaporator architecture. (d) Heat flux distribution and light reflection on the sidewalls with an external reflector (the reflector is a pure aluminum circular truncated cone). (e) Capillary‐driven water transport pathways through the interconnected pore network.

## 2 Results and Discussion

To verify the universality of this evaporator design strategy, we fabricated a PDMSE evaporator on a larger scale (Figure 2a). To investigate the microstructure of PDMSE, scanning electron microscopy (SEM) was performed. As shown in Figure 2b–e, PDMSE exhibits a well‐defined cylindrical 3D structure. SEM images (Figure 2f,g) further reveal a micrometer‐scale, porous skeleton formed by interwoven polymer networks on both the surface and interior. Detailed SEM images of PDMSE and PDMSE after 8 h of evaporation are shown in Figures S1 and S2. Notably, this 3D interpenetrating network does not undergo significant capillary shrinkage or structural collapse during dehydration, demonstrating exceptional structural stability. It is precisely this robust and highly interconnected continuous pore structure that not only provides sufficient capillary driving force for efficient water transport but also endows the PDMSE with an ultralow bulk density and excellent mechanical elasticity.

**FIGURE 2:**
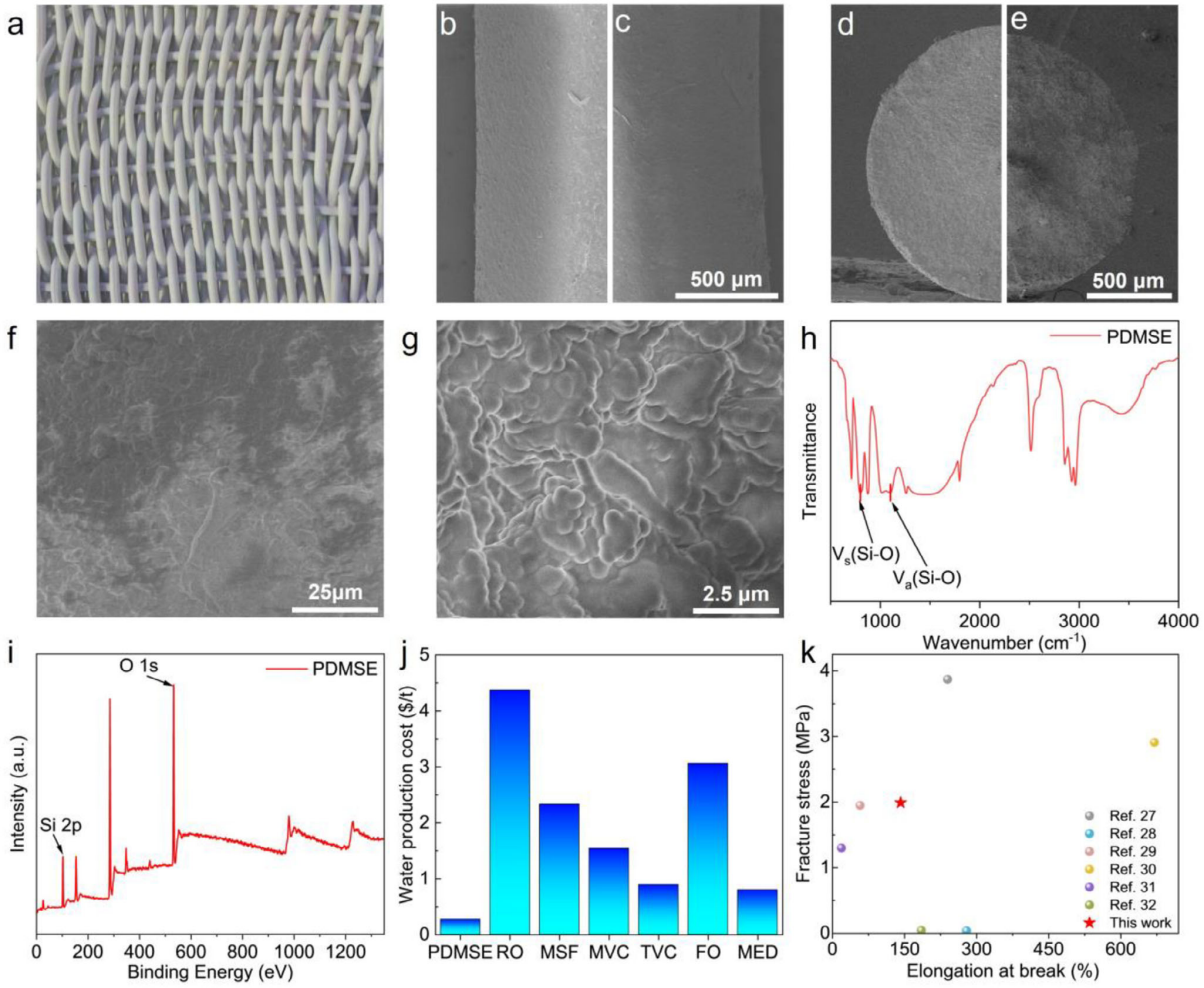
(a) Digital photos of the larger PDMSE. SEM images of the PDMSE. (b,d) Surface. SEM images of the PDMSE after 8 h evaporation in 3.5 wt.% brine under 1 sun illumination. (c,e,f,) Surface and (g) Cross‐sectional. (h) FT‐IR spectrum. (i) XPS survey spectrum. (j) Comparison of water production costs among different desalination technologies. (k) Comparison of maximum loads among various reported evaporator systems.

Following the elucidation of the physical porous structure, Fourier transform infrared (FTIR) spectroscopy and x‐ray photoelectron spectroscopy (XPS) were employed to investigate the chemical compositions and bonds of PDMSE. As shown in Figure 2h, the FTIR spectrum exhibits strong characteristic peaks at 800 and 1000–1100 cm^−1^. These peaks are attributed to the symmetric and asymmetric stretching vibrations of the Si─O─Si backbone, respectively, providing definitive evidence for the successful construction of a typical crosslinked network structure within the polydimethylsiloxane. Furthermore, XPS spectra were utilized to reveal the elemental composition of the material (Figure 2i). The strong binding energy peaks at 102.5 and 529.0 eV correspond to the characteristic signals of the Si 2p and O 1s orbital electrons, respectively. Additionally, the PDMSE fracture stress‐strain curve shown in Figure S3 demonstrates excellent mechanical stability. The high tear resistance and mechanical flexibility of the polyorganosiloxane network allow the evaporator to withstand repeated swelling‐dehydration cycles and mechanical disturbances without structural collapse. The above results directly verifying the fabrication of PDMSE with polyorganosiloxane. Finally, a comprehensive comparison of this work with recent reports on seawater desalination technology and advanced solar interface evaporators shows that PDMSE evaporators offer low water production costs and excellent mechanical strength, as shown in Figure 2j,k [21, 22, 23, 24, 25, 26, 27, 28, 29, 30, 31, 32].

Broadband light absorption and highly efficient thermal localization are fundamental prerequisites for achieving superior evaporation performance. Ultraviolet‐visible‐near‐infrared (UV–vis–NIR) spectroscopy demonstrates that PDMSE exhibits stable and acceptable light absorption characteristics (limiting absorbance > 60%, absorption rate of 17.67%) across a broad spectral range of 200–2500 nm (Figure 3a). Conventional black photothermal absorbers depend on high intrinsic absorption. In contrast, the white PDMSE uses a scattering‑dominated approach. Its three‑dimensional porous skeleton generates strong multiple scattering. This scattering prolongs the effective optical path and improves photon‐material interactions. The blue‐shaded region in Figure 3a represents the energy flux distribution of the AM 1.5G solar spectrum, highlighting the spectral overlap between the material's optical response and the maximum solar irradiance. Figure S4 shows the reflection spectrum of PDMSE. This overlap ensures efficient solar energy harvesting despite the material's white appearance. This excellent light absorption translates into a highly efficient photothermal response. To quantify this process, the transient heating behavior and spatial thermal field distribution of the evaporators were monitored under one‐sun illumination (1 sun) using thermocouples and an infrared (IR) camera, respectively. As shown in Figure 3b, within 10 min of illumination, the surface temperatures of both sample groups rapidly increase exponentially before reaching thermal equilibrium. PDMSE exhibits a low thermal conductivity (Figure S5) and maintains excellent thermal cycling efficiency over five thermal cycles (Figure S6). The intrinsically low thermal conductivity of the polyorganosiloxane matrix plays a critical role in confining the photothermally generated heat to the evaporative surface, minimizing conductive losses to the underlying bulk water and contributing to the efficient thermal localization observed in the temperature distribution profiles. The IR thermal images intuitively confirm the substantial contribution of the external reflector toward optimizing the 3D thermal field (Figure 3d). An annular aluminum foil reflector was coaxially positioned around the evaporator sidewalls. This configuration redirects scattered and transmitted light back onto the lateral surfaces, creating a more uniform 3D thermal field and enhancing sidewall heating. Upon 60 min of light irradiation, pure water reached a surface temperature of 26.0°C (Figure S7). The PDMSE, with its highly reflective white surface and intense diffuse reflection, showed limited light‐harvesting capacity in the absence of a reflector, yielding a peak surface temperature of merely 28.2°C. In contrast, upon introduction of the reflector, the surface temperature rapidly and uniformly increases to 34.5°C, which results from the secondary redirection of scattered light and the volumetric heating of the sidewalls of the sidewalls. This rapid temperature increase and efficient heat confinement at the evaporator surface provide a strong thermodynamic driving force for interfacial water vaporization.

**FIGURE 3:**
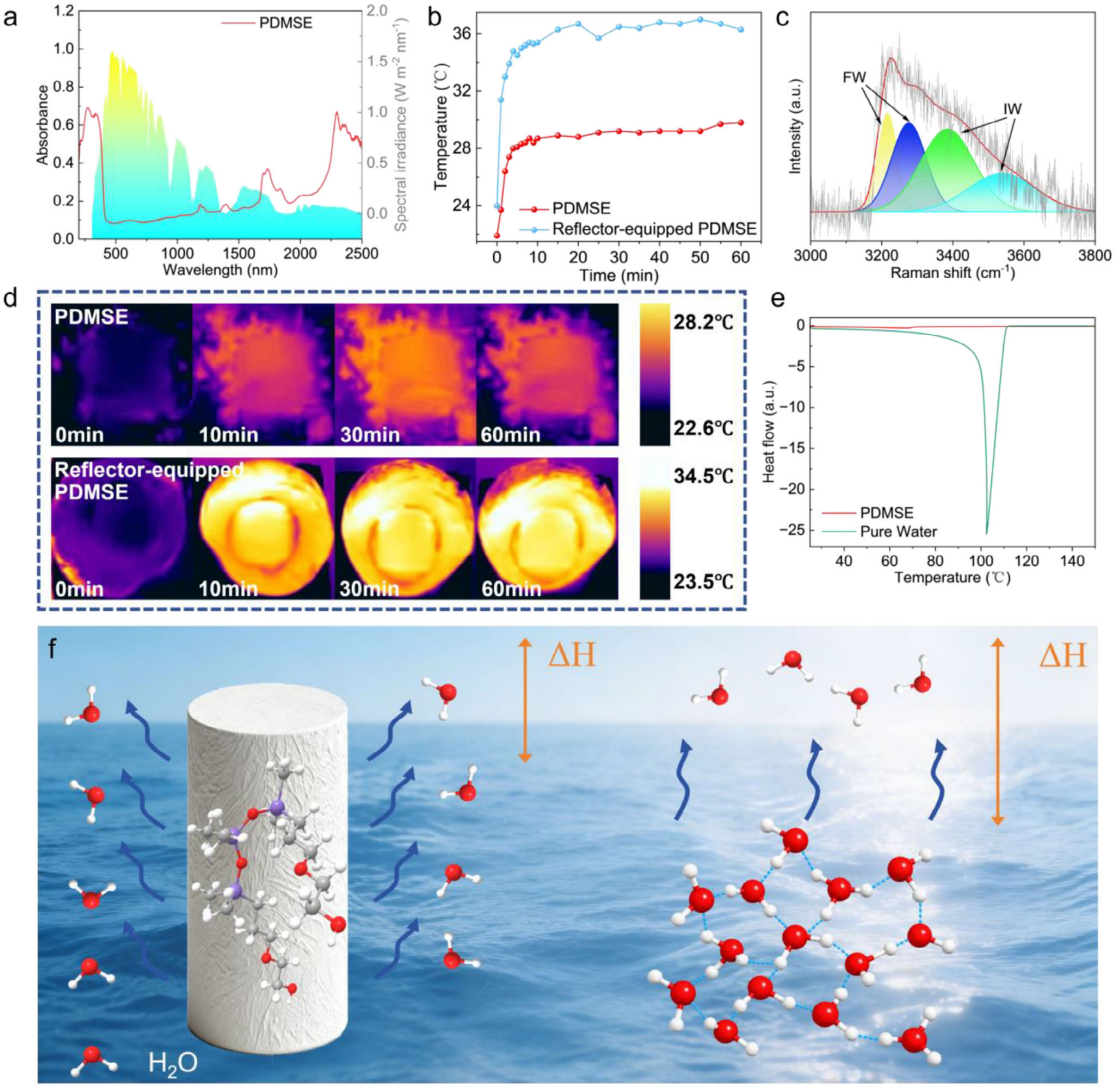
(a) UV–vis–NIR absorption spectrum of the PDMSE. (b) Temperature change at the top of the PDMSE evaporator placed on a water surface under 1 sun illumination. (c) Raman spectrum of the PDMSE. (d) Infrared thermal images showing the surface temperature change of the PDMSE evaporator within 10 min under 1 sun illumination. (e) DSC curves for pure water and water within the PDMSE. (f) Schematic of water state regulation in the PDMSE matrix.

Beyond maximizing interfacial heat input, reducing the vaporization enthalpy of water represents another strategy to further surpass the evaporation rate limit. In this regard, the unique polymer skeleton of PDMSE plays a critical role in regulating the state of water. Water contact angle measurements were performed to evaluate the surface wettability of PDMSE, which can be tailored through surface modification (Figures S8–S10). The pristine PDMSE exhibits intrinsic hydrophobicity, while hydrophilic and hydrophobic modifications yield distinct wettability states. These systematically varied surface properties demonstrate the tunable water affinity of the polyorganosiloxane platform, which plays a critical role in balancing capillary‐driven water supply and thermal localization during evaporation. Relying on the capillary force generated by its internal capillary channels, the material enables continuous upward delivery of salt water toward the evaporation interface. Meanwhile, its hydrophobic characteristic avoids extensive lateral spreading of water that would otherwise arise from overstrong hydrophilicity; such widespread water spreading would otherwise induce parasitic heat loss across the heat‐transfer layer, and the suppression of this adverse effect consequently elevates the photothermal evaporation efficiency. To investigate the binding states of water within PDMSE and their impact on evaporation performance, Raman spectroscopy analysis was conducted (Figure 3c). The characteristic peaks at 3216 and 3276 cm^−1^ are assigned to free water, while the peak at 3384 and 3538 cm^−1^ corresponds to interstitial water, which is characterized by weak hydrogen bonding [33]. Figure S11 shows the Raman fitting parameters. Calculations reveal that the ratio of intermediate‐state water (IW) to free water (FW) within the polymer network reaches 1.42. Such a high proportion of intermediate water indicates that the polar groups on the PDMSE surface effectively disrupt and weaken the strong hydrogen‐bonding interactions inherent among bulk water molecules. This molecular weakening reduces evaporation enthalpy significantly. Figure 3f shows schematic of water state regulation in the PDMSE matrix. The water state regulation in PDMSE does not rely on abundant hydrophilic functional groups. The polyorganosiloxane backbone contains methyl side groups and siloxane linkages. These groups are not strongly hydrophilic. However, they can interact with water molecules through weak van der Waals forces and hydrophobic hydration. At the polymer water interface, the hydrophobic segments create a confined environment that perturbs the tetrahedral hydrogen bonding network of bulk water. This effect promotes the formation of intermediate water species. The intermediate water has fewer hydrogen bonds per molecule and requires less energy to escape as vapor. The Raman spectra confirmed the existence of intermediate water with an IW to FW ratio of 1.42. This molecular level mechanism explains the reduced evaporation enthalpy without requiring strong hydrophilic sites. To more accurately estimate the enthalpy of evaporation in PDMSE, a dark evaporation experiment was performed, yielding a result of 987 J g^−1^, which is significantly lower than the theoretical value of 2450 J g^−1^. The original quality loss curves of pure water and PDMSE in a dark environment are shown in Figure S12. The PDMSE evaporator achieves a high evaporation efficiency of up to 90%, benefiting from the reduced energy requirement. As shown in Figure 3e, the heat flow signal of pure water drops sharply at the peak, indicating a rapid evaporation rate, whereas the heat flow signal of PDMSE changes slowly, suggesting that the evaporation behavior of water in PDMSE is different from that of pure water. However, DSC cannot accurately measure the thermokinetics during actual evaporation because it only measures the water stored in PDMSE, whereas PDMSE will continuously supply water through its unique structure during actual evaporation. Through the dual effects of enhanced interfacial thermal localization and reduced water vaporization energy consumption, the PDMSE evaporator successfully achieves highly efficient interfacial evaporation.

To evaluate the long‐term performance and salt resistance of the PDMSE evaporator, experiments were conducted under one standard sun illumination (1 sun) in a 3.5 wt.% NaCl solution. Figure 4a indicates that the steady‐state evaporation rate of the PDMSE system reaches 3.285 kg m^−2^ h^−1^. Benefiting from the external reflector, which further optimizes the spatial photothermal distribution, the evaporation rate of the reflector‐equipped PDMSE is further enhancing to 4.344 kg m^−2^ h^−1^. Furthermore, experimental observations reveal that 3D structured PDMSE delivers markedly enhanced evaporation performance relative to its 2D counterpart (Figure S13). The superior evaporation performance of the 3D PDMSE over its 2D counterpart primarily originates from two synergistic effects. First, the three‐dimensional columnar geometry provides additional sidewall surfaces for evaporation beyond the top face, substantially increasing the effective evaporation area. Second, the vertical sidewalls, when coupled with the external reflector, enable secondary light interception and scattering, which redirects diffuse solar radiation onto the lateral surfaces and promotes more uniform spatial heat distribution. In contrast, the 2D planar configuration is largely limited to top‐surface absorption and suffers from greater convective heat loss to the ambient air due to the absence of sidewall thermal confinement. These architectural advantages collectively account for the markedly enhanced evaporation performance of the 3D design. This remarkable performance enhancement demonstrates the critical role of the reflector in 3D photothermal management and energy augmentation.

**FIGURE 4:**
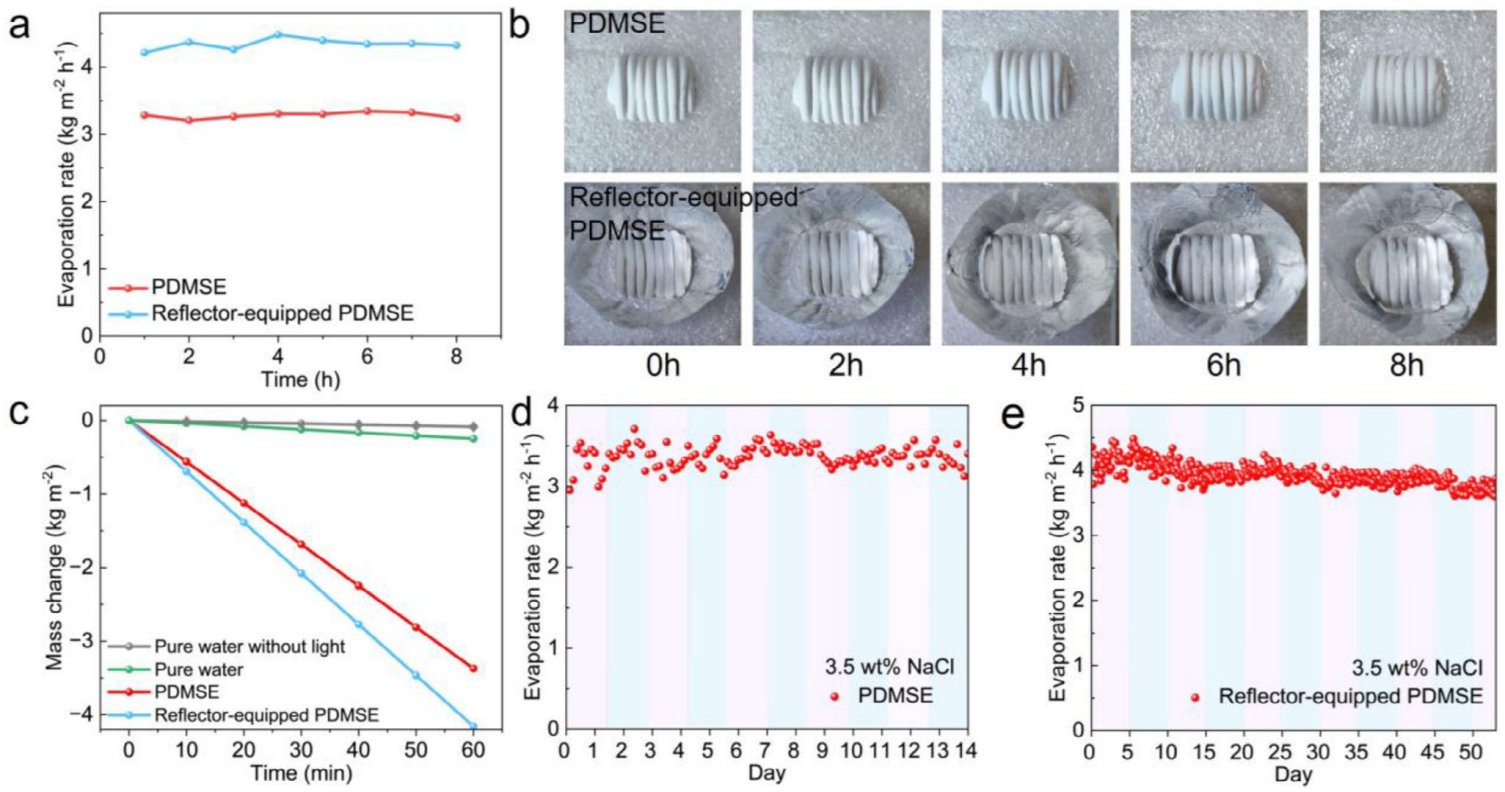
(a) Evaporation rates of the PDMSE and the reflector‐equipped PDMSE over time. (b) Digital photographs of the PDMSE and reflector‐equipped PDMSE surfaces after long‐term operation. (c) Comparison of mass change for the PDMSE, reflector‐equipped PDMSE, and pure water under illuminated and dark conditions. (d) Cyclic stability test of the PDMSE. (e) Cyclic stability test of the reflector‐equipped PDMSE.

For practical seawater desalination applications, the salt‐crystallization resistance of an evaporator during the continuous processing of high‐concentration brines is a metric that dictates its operational lifespan. Therefore, extreme stability tests were performed by continuously floating both groups of PDMSE evaporators on a 3.5 wt.% NaCl solution to simulate seawater (operating for 8 continuous hours per day). As confirmed by the photographs in Figure 4b, the material surface remains consistently clean throughout the 8 h high‐intensity evaporation process, with no macroscopic salt accumulation observed. This anti‐fouling capability is attributed to the highly developed, 3D interconnected pore network within the PDMSE, which leverages concentration gradients to achieve rapid ion diffusion and convective exchange, thereby spontaneously rejecting the concentrated brine accumulated at the surface. As depicted in Figure 4c, the cumulative water evaporation of both the PDMSE and the reflector‐equipped PDMSE exhibits excellent linear growth under illumination, significantly outperforming the natural evaporation of pure water. Under different lighting conditions, the quality changes of PDMSE, PDMSE with reflectors, pure water, and pure water with reflectors were compared (Figure S14) to distinguish the contribution of PDMSE itself from the heating effect caused by the reflectors. More remarkably, the long‑term cycling data (Figure 4d,e) indicate that after 14 and 53 days high‑salinity runs, the daily evaporation rates for both PDMSE configurations remain stable. There is no sign of decay or degradation in either system. When immersed in seawater, PDMSE showed no degradation after one hundred ninety‐seven days, demonstrating excellent stability and corrosion resistance (Figure S15). The sustained evaporation performance over 53 days of cyclic operation and the intact structural integrity after 197 days of seawater immersion directly arise from the intrinsic chemical inertness of the polyorganosiloxane backbone. The strong Si‐O‐Si crosslinked network provides resistance against hydrolysis, oxidation, and microbes. These factors usually damage evaporators based on biomass or hydrogels. Consequently, this network supports extended operation, and its material remains intact over time. Figures S16–S20 present the SEM images, FTIR and XPS spectra, fracture stress‐strain curve, and water contact angles of PDMSE after immersion in real seawater. Compared with the pristine sample, no significant changes were observed in any of these characterizations, confirming the chemical stability and corrosion resistance of the polyorganosiloxane matrix. The PDMSE evaporator exhibits excellent photothermal evaporation performance and outstanding operational durability. These findings highlight its strong potential for desalination applications.

In practical desalination applications involving high‐concentration NaCl solution, rapid water vaporization can easily drive the salt concentration at the evaporation interface to a supersaturated state. The subsequent salt crystallization not only increases solar reflectance but also obstructs water transport channels, leading to a degradation in evaporation performance. To investigate the dynamic salt‐rejection mechanism of the PDMSE evaporator, solid salt was artificially placed at the interface, and its dissolution and transport processes were monitored in real time. Figure 5a shows that under one sun illumination, the interconnected porous capillary network in the PDMSE generates a strong concentration‑gradient driving force. The most pronounced change occurs within 3 min, during which the bulk of the surface salt dissolves. Complete dissolution is achieved within 7 min for the reflector‐free PDMSE, whereas the reflector‐equipped configuration reaches full dissolution in 5 min, as indicated by the disappearance of the white salt residues at the final time points. The marked difference between the 2 min and final states in each series clearly demonstrates the rapid salt rejection, while the enhanced performance with the reflector is most evident by comparing the 5 min time points between the two configurations. This photothermally enhanced salt‐rejection phenomenon is primarily attributed to the elevated local temperatures induced by the reflector, which effectively reduce fluid viscosity and significantly enhance the ion diffusion coefficient. As a control, the salt‐rejection test conducted in a dark environment reveals that the complete migration of salt requires over 10 min (Figure 5b). The hydrophobic nature of the silicone surface, characterized by its low surface energy, effectively prevents salt crystal adhesion and pore blockage, thereby ensuring uninterrupted water transport and vapor escape. This surface property, combined with the porous capillary network, enables the rapid back‐diffusion of concentrated brine observed in the salt dissolution tests. This further confirms that although the intrinsic porous structure of PDMSE serves as the foundational basis for spontaneous salt rejection, the light‐driven localized thermocapillary convection significantly accelerates this mass transfer process.

**FIGURE 5:**
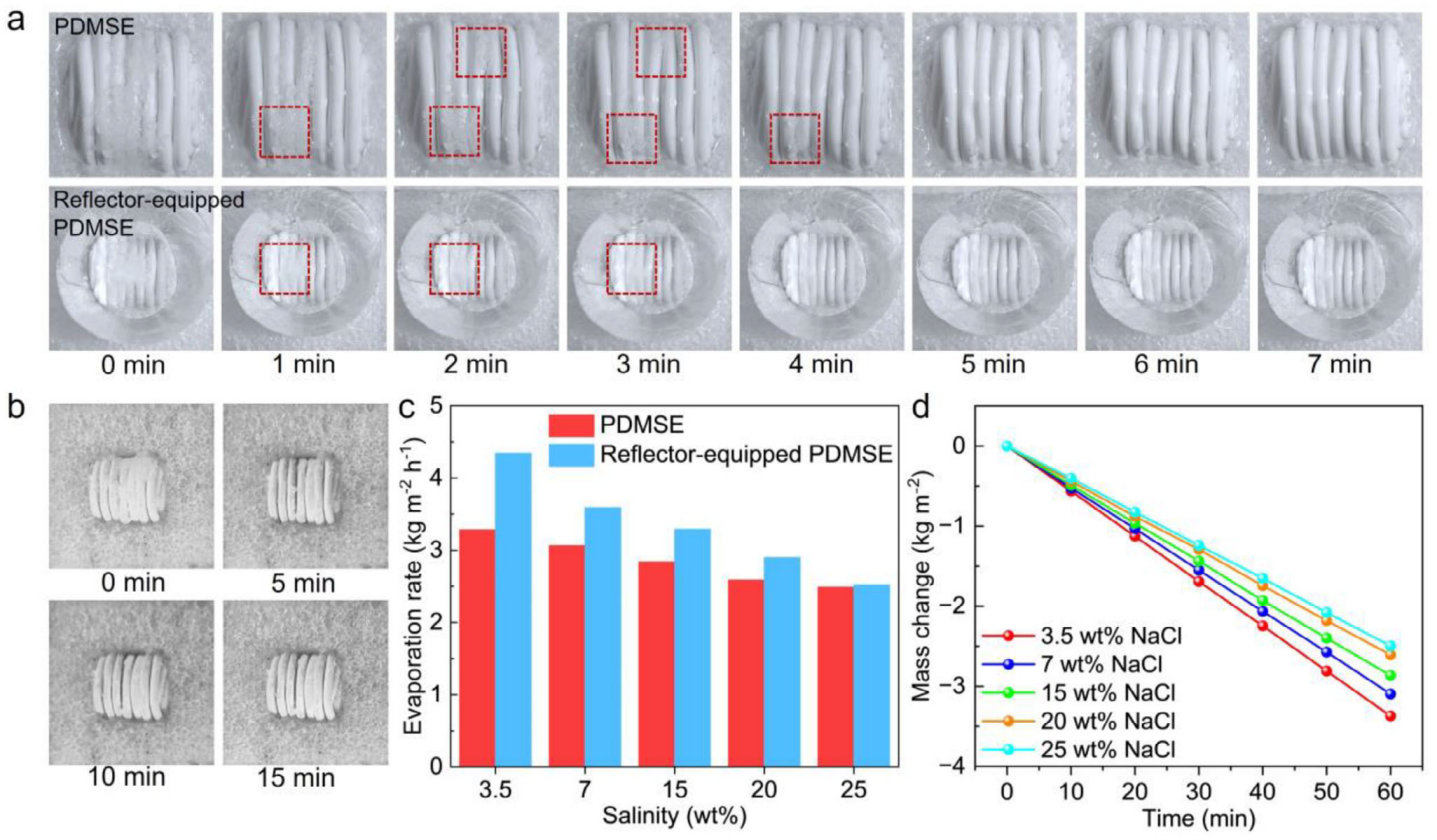
(a) Salt dissolution tests for the PDMSE and the reflector‐equipped PDMSE under 1 sun illumination. (b) Salt dissolution test for the PDMSE in the dark. (c) Evaporation rates of the PDMSE and the reflector‐equipped PDMSE over time at different salinities. (d) Comparison of mass change for the PDMSE at different salinities.

Based on the aforementioned excellent anti‐fouling and salt‐rejection mechanisms, the operational limits of the evaporator under various extreme salinities were systematically evaluated. As shown in Figure 5c,d, as the NaCl mass fraction increases from 3.5 to 25 wt.%, the steady‐state evaporation rate of the PDMSE decreases from 3.285 to 2.488 kg m^−2^ h^−1^. Meanwhile, the reflector‐equipped PDMSE consistently maintains superior evaporation performance, with its rate dropping from 4.344 to 2.522 kg m^−2^ h^−1^. Although the overall rates exhibit a slight downward trend, governed by the thermodynamic principle that high concentrations of ions restrict the volatilization of water molecules, none of the tested samples experienced crystallization under extreme salinities. This demonstrates a highly competitive capability for continuous and stable desalination. These results prove that the PDMSE evaporator possesses exceptional multi‐salinity adaptability and extremely robust anti‐crystallization capabilities, highlighting its tremendous potential for processing real‐world, high‐concentration brines.

Beyond its remarkable seawater desalination efficiency, this work further evaluated the purification potential of PDMSE in industrial wastewater containing complex organic pollutants. Purification tests were conducted using Rhodamine B (RhB, 20 mg L^−1^) and methyl orange (MO, 20 mg L^−1^) as models. As shown in Figure 6a,b, the original solutions exhibited strong characteristic UV–vis absorption peaks at 554 and 464 nm, respectively. However, these characteristic peaks completely disappeared in the pure water collected following PDMSE‐enabled evaporation and condensation. This result confirms that, driven by the photothermal phase‐change mechanism, water molecules efficiently escape as pure water vapor. Meanwhile, macromolecular dyes and non‐volatile pollutants are entirely retained on the water‐supply side, demonstrating the exceptional performance of PDMSE in treating complex organic wastewater. As illustrated by the comparative analysis in Figure 6e, PDMSE maintained highly stable evaporation performance whether treating MO solutions, RhB solutions, or real seawater. Furthermore, the reflector‐equipped PDMSE evaporator achieves a high evaporation rate in 3.5 wt.% brine solutions at both pH = 3 and pH = 10, and simultaneously exhibits pollutant adsorption capacity (Figures S21 and S22). These results demonstrate that the PDMSE evaporator can operate stably under extreme pH conditions and possesses a certain seawater purification capability. Particularly, the system with a reflector achieved notably higher evaporation rates than the one without, regardless of water quality. Moreover, it displayed strong fouling resistance and versatile applicability to different water sources.

**FIGURE 6:**
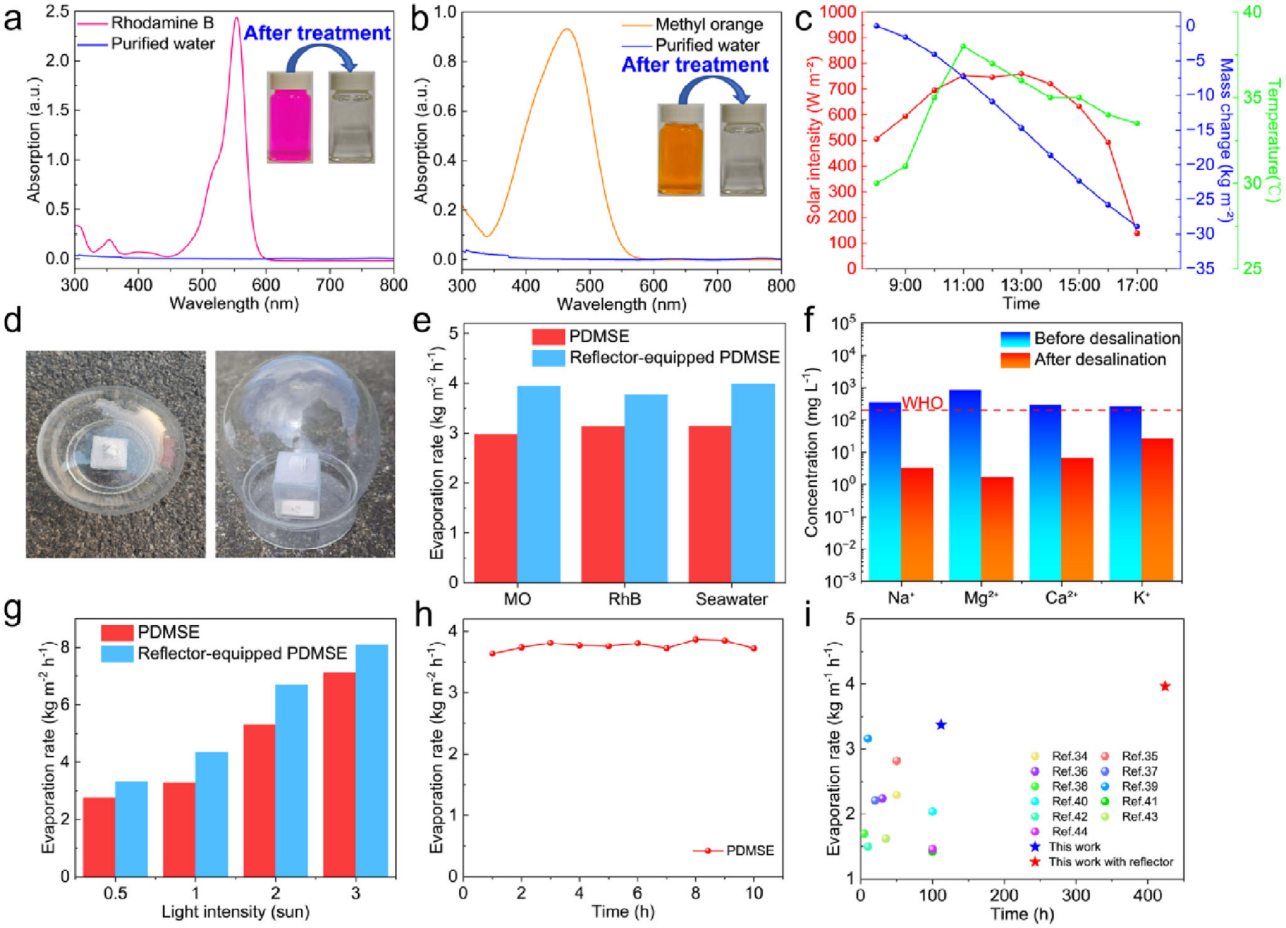
(a) UV–vis absorption spectra of the RhB solution and (b) the MO solution before and after evaporation. (c) Separately measured mass change of the PDMSE, solar irradiance, and ambient temperature during outdoor operation. (d) Side and top views of the PDMSE evaporation setup. (e) Evaporation rates of the PDMSE in different solutions. (f) Concentrations of Na^+^, K^+^, Mg^2+^, and Ca^2+^ ions before and after seawater desalination. (g) Evaporation rates of the PDMSE and reflector‐equipped PDMSE under different light intensities. (h) Evaporation rate of the PDMSE tested in real seawater. (i) Comparison of water evaporation rate and cycle time among various reported evaporator systems.

To verify the application of the PDMSE evaporator, an outdoor solar evaporation and condensate collection system was constructed, as shown in Figure 6d. Figure 6c comprehensively records the dynamic evolution of solar irradiance and the corresponding mass change in cumulative freshwater yield during the daytime (8:00 to 18:00) on October 7, 2025. The water evaporation rate of the system was highly synchronized with the diurnal fluctuations of natural light intensity. Furthermore, during continuous outdoor operation, no obvious salt accumulation was observed on the PDMSE surface, confirming its anti‐crystallization stability under naturally fluctuating heat sources. Organic polymers suffer from photo‑oxidative degradation when exposed to sunlight over time. Polyorganosiloxane avoids this problem. It offers inherent resistance to UV and ozone. Hence, it delivers stable photothermal performance outdoors. Its structure does not degrade. Moreover, it does not rely on any protective coatings or stabilizers. More importantly, the concentrations of key ions (Na^+^, Mg^2+^, Ca^2+^, and K^+^) in both the real seawater and the collected condensate were precisely measured using inductively coupled plasma optical emission spectrometry (ICP‐OES) (Figure 6f). Following desalination, the concentrations of all respective ions exhibit a decrease of 1 to 2 orders of magnitude, fully satisfying the drinking water safety standards mandated by the WHO and directly confirming the practical value of the device in providing safe drinking water.

To explore the operational potential of the evaporator under extreme energy input, the response of the device to various light intensities was systematically tested (Figure 6g). As the light intensity increased from 0.5 sun to 3.0 sun, the evaporation rate of the PDMSE climbed from 2.751 to 7.113 kg m^−2^ h^−1^. Particularly striking is that the reflector‐equipped system further amplified its spatial light‐harvesting advantage under intense illumination, achieving an impressive maximum evaporation rate of 8.087 kg m^−2^ h^−1^ at 3.0 sun. High light intensity not only provides a more abundant interfacial thermodynamic driving force but also significantly accelerates the desorption and escape of vapor. Long‐term cycling performance tests (Figure 6h) reveal that PDMSE consistently maintained a stable evaporation rate of 3.769 kg m^−2^ h^−1^ during continuous high‐intensity operation, with no observable performance degradation. The evaporation rate of reflector‐equipped PDMSE is marginally enhanced following the introduction of carbon black (Figure S23). Finally, a comprehensive comparison between this work and advanced solar interfacial evaporators reported in recent literature reveals that the PDMSE evaporator possesses truly exceptional solar evaporation performance, as depicted in Figure 6i [34, 35, 36, 37, 38, 39, 40, 41, 42, 43, 44].

## 3 Conclusion

In summary, a polyorganosiloxane‐based interfacial solar evaporator coupled with external optical management was successfully constructed. The chemically inert Si─O─Si crosslinked porous network endows PDMSE with outstanding structural stability and inherently low thermal loss. The introduction of an external reflector overcomes the light‐harvesting limitations of traditional two‐dimensional planes and enables deep localization of spatial heat flux. Meanwhile, the polar polymer skeleton disrupts the hydrogen bonding network of water molecules, yielding the ratio of intermediate‐state water (IW) to free water (FW) of 1.42 and substantially reducing the vaporization enthalpy at the thermodynamic source. As a result, the reflector‐equipped evaporator achieves remarkable evaporation rates of 4.344 kg m^−2^ h^−1^ under 1 sun and 8.087 kg m^−2^ h^−1^ under 3 suns. It operates robustly in near saturated brines up to 25 wt.% and maintains without degradation cyclic operation in 3.5 wt% simulated seawater for 53 days. From a practical perspective, the PDMSE evaporator is compatible with scalable manufacturing routes such as template‐assisted foaming and spray coating, with raw material costs comparable to those of other reported evaporator systems. While large‐area fabrication may introduce minor performance losses due to pore structure inhomogeneity or reduced geometric precision, the exceptional durability demonstrated in this work (53 days cycling, 197 days seawater immersion) suggests that core performance metrics are expected to remain robust upon scale‐up with proper process optimization. This research provides a highly scalable polyorganosiloxane‐based for high salinity industrial wastewater treatment and extreme environment water supply, advancing next generation sustainable water harvesting technologies.

## Author Contributions

**Jiaxian Zheng**: investigation, writing – review and editing, conceptualization. **Liqiang Zhang**: investigation, writing – original draft. **Haoqi Ni**: formal analysis. **Shuozhen Chen**: methodology, data curation. **Xiangfeng Lin**: project administration, formal analysis, data curation, methodology. **Liwei Wang**: conceptualization, funding acquisition, validation, supervision. **Zhanhui Yuan**: conceptualization, methodology, software, data curation, supervision, resources, project administration, formal analysis, validation, investigation, funding acquisition, writing – review and editing.

## Conflicts of Interest

The authors declare no conflicts of interest.

## Supporting information




**Supporting File**: advs78278‐sup‐0001‐SuppMat.docx.

## Data Availability

The data that supports the findings of this study are available in the supplementary material of this article.
