# Supplementary material for: Long‐Lifespan Polyorganosiloxane‐Based Silicone Evaporator for High‐Efficiency Seawater Desalination
Source: Adv Sci (Weinh). 2026 Oct 14:e78278. Online ahead of print. doi: 10.1002/advs.78278 (PMC13669206; doi:10.1002/advs.78278)
Supplement: Supplementary file 1 [file ADVS-9999-e78278-s001.docx]

Supporting Information

Long-Lifespan Polyorganosiloxane-Based Silicone Evaporator for High-Efficiency Seawater Desalination

*Jiaxian Zheng,^1^ Liqiang Zhang,^1^ Haoqi Ni,^1^ Shuozhen Chen, Xiangfeng Lin,* Liwei Wang*, and Zhanhui Yuan**

J. Zheng, L. Zhang, H. Ni, S. Chen, X. Lin, and Z. Yuan

College of Materials Engineering, Fujian Agriculture and Forestry University, Fuzhou 350108, P.R. China

E-mail: xiangfenglin@fafu.edu.cn; zhanhuiyuan@fafu.edu.cn

H. Ni and L. Wang

College of Materials and Chemical Engineering Minjiang University, Fuzhou, Fujian 350108, China E-mail: [wlw@mju.edu.cn](mailto:wlw@mju.edu.cn)

*^1^* These authors contributed equally to this work.

**Chemicals and materials**

The commercial silicone sealant (Jia Yun) was purchased from Shenzhen Muyaju Bathroom Co., Ltd. Its main component is one-component silicone adhesive, which is a paste-like material primarily made of polydimethylsiloxane. By filling the commercial silicone sealant into a 20 ml syringe (with a needle specification of an outer diameter of 2 mm and an inner diameter of 1.8 mm) and extruding it at a constant speed, the initial sample can be obtained after natural air drying for 24 hours. Then, the initial sample is wrapped and curled into a coil-shaped evaporator with dimensions of 1 cm $\text{×}$1 cm $\text{×}$1 cm to obtain the PDMSE evaporator. Carbon black modified PDMSE solar evaporator, prepared from commercial silicone sealant via carbon black modification at a carbon-to-sealant ratio of 1 mg : 1 mL. The purpose of adding carbon black was to evaluate the effect of enhanced light absorption on the evaporation performance. Rhodamine B was obtained from Shanghai Aladdin Biochemical Technology Co., Ltd. The insulating foam was sourced from the local market in Fuzhou. Sodium chloride (NaCl), sodium hydroxide (NaOH), hydrochloric acid (HCl), and methyl orange were all supplied by Sinopharm Chemical Reagent Co., Ltd.

**Characterizations**

The micro-morphological features of the samples were characterized using scanning electron microscopy (SEM, Hitachi SU8010). The sample preparation process was as follows: For surface morphology observation, the vacuum-dried sample was fixed onto a flat sample stage using conductive adhesive. For cross-sectional morphology observation, the sample was first freeze-fractured in liquid nitrogen to obtain a clean, flat surface, followed by vacuum drying. The prepared cross-section was then fixed onto a dedicated sample stage using conductive adhesive. To enhance conductivity, all samples were sputter-coated with a thin gold layer prior to imaging to ensure clear microscopic images. The optical absorption properties were characterized using a UV-vis-NIR spectrophotometer (Lambda 750S). Prior to measurement, the instrument was calibrated with a standard white reference to ensure data accuracy. The wavelength range was set from 200 to 2500 nm. The surface wettability of the materials was quantitatively characterized using a contact angle goniometer (DSA 30, range: 0-180°). The prepared sample was fixed onto the sample stage, and its position was adjusted for measurement. The chemical functional groups of the samples were characterized by Fourier transform infrared spectroscopy (FT-IR, Nicolet 7000) over a wavenumber range of 400 to 4000 cm⁻¹.The state of water within the evaporator was quantitatively analyzed using Raman spectroscopy (LabBRAM HR Evolution, France). A 532 nm laser was used as the excitation source, with spectra collected in the range of 1000-4000 cm^-1^. Through Gaussian peak fitting, the relative content of intermediate water and free water was calculated based on the integrated area ratio of their characteristic peaks. At least five different areas on each sample were tested to ensure data representativeness. The concentration changes of major cations (Na^+^, K^+^, Ca^2+^, Mg^2+^) before and after desalination were quantitatively analyzed using inductively coupled plasma optical emission spectrometry (ICP-OES, Agilent 5110). The concentrations of these four ions were measured to assess whether the produced water met drinking water standards. Differential scanning calorimetry (DSC) experiments were conducted under the following conditions: samples were heated from an initial temperature of 25°C to a final temperature of 200°C at a linear heating rate of 5°C min^-1^. This method facilitated a detailed analysis of the water evaporation enthalpy and phase transitions within the sample. The comprehensive dataset obtained through the aforementioned characterization methods provides robust support for the excellent performance of the organic polysiloxane as a photothermal conversion material.

Evaporation experiments were conducted under simulated solar illumination. A xenon lamp light source (CEL-65 HXF300) was used to simulate sunlight, and its intensity was calibrated to 1 kW m^-2^ (equivalent to 1 sun) using an optical flux meter (CEL-NP2000-2). The PDMSE evaporator was placed on a high-precision electronic balance (FA1004, accuracy: 0.0001 g) for testing under controlled ambient conditions (25°C, 50% relative humidity). The mass change was recorded in real-time.

The evaporation rate (m, in kg m^-2^ h^-1^) was calculated according to Equation 1:

$\text{m=Δm/(A×t)}$	(1)

where Δm is the mass change of water (kg), A is the evaporation area (m^2^), and t is the evaporation time (h).

The equivalent enthalpy of evaporation (Δ_vap_H_m_(equ)) for the evaporator was determined using the dark evaporation method reported in the literature. Specifically, the evaporation amounts of the solar evaporator and bulk water with an identical surface area were measured separately under dark conditions, at ambient pressure and room temperature (25°C). Assuming identical energy input from the environment to both systems, the equivalent enthalpy of evaporation for water within the PDMSE was calculated based on the principle of energy conservation, as expressed in Equation 2:

$\text{E}_{\text{in}}\text{=}\text{Δ}_{\text{vap}}\text{H}_{\text{m}}\text{(}\text{equ}\text{)×}\text{m}_{\text{g}}\text{=}\text{Δ}_{\text{vap}}\text{H}_{\text{m}}\text{(}\text{0}\text{)×}\text{m}_{\text{0}}$	(2)

Here, E_in_ represents the total energy absorbed per hour from the environment; m_0_ and m_g_ denote the mass changes of pure water and the PDMSE evaporator, respectively, over one hour; Δ_vap_H_m_(0) is the evaporation enthalpy of pure water; and Δ_vap_H_m_(equ) is the equivalent evaporation enthalpy of the PDMSE evaporator.

The photothermal evaporation efficiency (η) of the PDMSE was calculated using Equation 3:

$\text{η=[m×}\text{Δ}_{\text{vap}}\text{H}_{\text{m}}\text{(equ)]/}\text{P}_{\text{0}}\text{×100\%}$	(3)

where m is the evaporation rate under 1 sun illumination, Δ_vap_H_m_(equ) is the equivalent evaporation enthalpy, and P_0_ is the incident solar power density corresponding to 1 sun intensity (1 kW m^-2^).

Neglecting optical losses including reflection loss, light leakage, and stray light loss, and normalizing the actual optical power incident on the evaporator surface to unity, the photothermal evaporation efficiency (η) of PDMSE is calculated according to Equation 4:

$\text{η=[m×}\text{Δ}_{\text{vap}}\text{H}_{\text{m}}\text{(equ)]/[}I_{0}\cdot A_{\mathrm{ap}}\text{]}$	(4)

where m is the evaporation rate under 1 sun illumination (kg h^-1^), Δ_vap_H_m_(equ) is the equivalent evaporation enthalpy, I_0_ is the incident solar irradiance on the aperture area of the evaporator and A_ap_ is the effective aperture illuminated area of the evaporator. The incident light is directed vertically downward onto the evaporator top surface. The diameter of the reflector is 2.25 cm. The effective aperture illuminated area of the evaporator used for efficiency calculation is the physical area of PDMSE plus the area of the side walls.

Without considering scattering loss,the average absorption rate ($\bar{\alpha}$) of PDMSE was calculated using Equations 5 and 6.

$\alpha\left( \lambda\right)=1-T\left( \lambda\right)-R\left( \lambda\right)$	(5)

$\bar{\alpha}=\left[ \int_{200}^{2500} \alpha\left( \lambda\right)\times I_{AM_{1.5G}}\left( \lambda\right)ⅆ\lambda\right]/\left[ \int_{200}^{2500} I_{AM_{1.5G}}\left( \lambda\right)ⅆ\lambda\right]$	(6)

where α(λ) denotes the spectral absorptance at wavelength λ, T(λ) denotes the spectral transmittance at wavelength λ, R(λ) denotes the spectral reflectance at wavelength λ, and I_AM1.5G_(λ) corresponds to the standard AM1.5G solar irradiance spectrum.

**Figure S1.** SEM images of the PDMSE. a) Surface and b, c) Cross-sectional.

**Figure S2.** SEM cross-sectional images of the PDMSE after 8-hour evaporation in 3.5 wt% brine under 1 sun illumination.

**Figure S3.** Fracture stress-strain curve of PDMSE.

**Figure S4.** The reflection spectrum of PDMSE.

**Figure S5.** Thermal conductivity of the PDMSE.

**Figure S6.** Photothermal stability of PDMSE evaluated over five consecutive heating-cooling cycles.

**Figure S7.** Temperature variation of pure water.

**Figure S8.** Water contact angle of the PDMSE.

**Figure S9.** Water contact angle of the PDMSE after hydrophilic modification.

**Figure S10.** Water contact angle of the PDMSE after hydrophobic modification.

**Figure S11.** The Raman spectrum of PDMSE (with the Raman fitting parameters supplemented).

**Figure S12.** The original mass loss curves of PDMSE under dark conditions.

**Figure S13.** Mass change of water involving PDMSE-2D and PDMSE in 3.5 wt% brine under 1.0 kW m^−2^ irradiation.

**Figure S14.** Comparison of quality changes of PDMSE, PDMSE equipped with a reflector, pure water, and pure water equipped with a reflector under light conditions.

**Figure S15.** PDMS were immersed in real seawater for 197 days with initial digital images.

**Figure S16.** SEM images of PDMSE after immersion in real seawater. a-c) Surface and d, e) Cross-sectional.

**Figure S17.** FT-IR spectrum of PDMSE after immersion in real seawater.

**Figure S18.** XPS survey spectrum of PDMSE after immersion in real seawater.

**Figure S19.** Fracture stress-strain curve of PDMSE after immersion in real seawater.

**Figure S20.** Water contact angle of PDMSE after immersion in real seawater.

**Figure S21.** Evaporation rate of PDMSE(with reflector) in 3.5 wt% brine with pH = 3 and pH = 10.

**Figure S22.** Pollutant adsorption rate of the PDMSE.

**Figure S23.** Evaporation rate of PDMSE with carbon black.
